# Self-Help and Charitable Aid

**Published:** 1896-01-11

**Authors:** 


### Modern Sociology.

### SELF-HELP AND CHARITABLE AID.

The changes and chances of this mortal life are so varied, so fluctuating are the ups and downs of fortune, that there are few who can regard themselves at any time as absolutely secure from want in the future. The millionaire may, it is true, look forward to immunity from times of poverty, but with the majority this is not so. The larger number of the professional and middle classes cannot afford to live without reasonable precautions against a possible rainy day. It is one of the results of improved education that it inculcates the habit of looking forward. Those who have no thought beyond the passing hour are generally the uncultured. Nemesis, as a rule, follows neglect of the dictates of prudence. Failure in business, or the bed of sickness, may temporarily give rise to straitened circumstances. Thus arises the necessity for a measure of self-help on the part of nearly all sections of the community, and for provision against future contingencies. If this is true with regard to the middle classes, much more is it the case with the humbler members of the commonwealth.

The labourer with his twenty-three shillings a week is in a measure forced to live from hand to mouth. We shall not be doing him any service in the long run unless we afford him encouragement to help himself in the hours of emergency without constantly turning to others to lift him out of the slough into which he has temporarily fallen. Let us imagine the case of a weekly wage-earner laid on the shelf for a while, and incapacitated from following his daily avocation There are many agencies to whom he may apply for assistance. He may turn to the minister of religion, or to one of the numerous societies which lay themselves out to deal with cases of this description. It will largely depend upon the methods adopted by those to whom application is first made whether the man will henceforth drift into the habit of constantly looking to others for help at critical periods, or having learnt a pregnant lesson will trust to his own efforts in future difficulties.

Those whose work lies amongst the poor cannot but be dismayed at the manner in which numbers of this class will rush to avail themselves of the alms of the charitable, stretching out a hand in every direction where there is anything to be gained. If a man who has embarked on this course does not learn a salutary warning at the beginning of his career, if the necessity of self-reliance and forethought is not impressed upon him by those to whom he applies, the chances are that he will degenerate into the loafer or writer of begging letters, and, abandoning all honest work, become merely a parasite upon society.

It cannot, we think, be denied that many of the socialist schemes, now so popular, err in this direction and tend to undermine all self-reliance. They foster the idea that the individual citizen is to turn to the State in all times of distress rather than to depend on his own exertions. The State, it is urged, is to look after his children, however numerous they may become; the State is to furnish him with work in times of slackness, and eventually, when old age has overtaken him, is to provide a pension for his declining years. But how is the State itself to be robust and healthy unless it is composed of a body of self-reliant, sturdy, and independent citizens? We cannot, then, too strenuously urge upon all those who have anything to do with the administration of relief to avoid any step which is likely to destroy the self-respect and self-reliance of the recipients of their charity.

It is almost hopeless to preach the necessity of providence and forethought when multitudinous agencies are at work, the result of whose operations tends in a contrary direction to that here indicated. Of course, we are not deprecating the uses of charity, we are merely insisting that the truest charity will not always of necessity entail almsgiving. The heart so easily outruns the head, and men are cruel meaning to be kind. It is easier to toss a beggar a copper in the street than zealously to inquire into his condition and endeavour to place him again permanently on his feet. But let him who in so acting, would congratulate himself that he has done a charitable action, lay no such flattering unction to his soul. For the recipient of his charity will be no whit permanently relieved, but rather encouraged to continue his trade until all inducements to self-help are removed. Let the would-be charitable endeavour to make himself personally acquainted with the circumstances of the case, set himself to ascertain what are the causes which have brought about the man's present unhappy condition, and so, by a right understanding of the facts, bring it eventually to a lasting and successful issue. If he has no time at his disposal himself to spare for thus thoroughly dealing with the case, his best course will be to put it into the hands of some society, of the wisdom of whose methods he is assured.

Commonplace though it is, this lesson cannot be too often insisted upon as long as the majority of the charitably disposed are still found to ignore it. Before all things, our endeavour must be to look upon the man whom we would assist as a fellow and a brother. Nothing must be done, then, to impair his self-respect, or to foster the belief that there will at all times be found others ready to come to his rescue. In times of sickness, it is true, the rigour of our rule may be somewhat relaxed; but even here it will be well to point out to the sick man the evils of not belonging to a provident club, and to urge him with returning health to repair the omission, or even to make help conditional upon joining some friendly society. When the sufferer has done what his means reasonably permit towards helping himself, then only may extraneous aid be afforded without carrying with it the degradation of the recipient. Illness comes at times to all alike, and none can entirely dispense with skilled medical advice. Here, then, at any rate, is one opportunity of showing prudence, at present too much neglected. But whether it be in sickness or in health that relief may be administered, to "help others to help themselves" should ever be the motto of the truly charitable.

